# Consensus on potential interventions for improving glycaemic control among patients with type 2 diabetes in Kinshasa, Democratic Republic of the Congo: a Delphi study

**DOI:** 10.1080/16549716.2023.2247894

**Published:** 2023-08-25

**Authors:** Jean-Pierre Fina Lubaki, Olufemi Babatunde Omole, Joel Msafiri Francis

**Affiliations:** a Department of Family Medicine and Primary Care, Faculty of Health Sciences, University of the Witwatersrand, Johannesburg, South Africa; b Department of Family Medicine and Primary Care, Protestant University of the Congo, Kinshasa, Democratic Republic of the Congo

**Keywords:** Policy, consensus, Delphi, interventions, glycaemic control, type 2 diabetes

## Abstract

**Background:**

Poor glycaemic control is a multifactorial and complex problem with dire clinical and economic implications. In the Democratic Republic of the Congo, recent studies have shown alarming poor control rates. There is no policy framework to guide corrective actions.

**Objectives:**

To build a consensus on interventions to improve glycaemic control among patients with type 2 diabetes in Kinshasa, Democratic Republic of the Congo.

**Methods:**

This was a two-round electronic Delphi study involving 31 local and 5 international experts. The experts rated proposed interventions from previous studies on glycaemic control in sub-Saharan Africa and Kinshasa on a 4-Likert scale questionnaire. Additionally, the experts were asked to suggest other recommendations useful for the purpose. The mode, mean and standard deviation of each statement were calculated for each round.

**Results:**

Participants reached consensus in five domains that included 39 statements on how to improve glycaemic control in Kinshasa: strengthening the health system, enhancing the awareness of diabetes, alleviating the financial burden of diabetes, enhancing the adoption of lifestyle modifications, and reducing the proportion of undiagnosed diabetes.

**Conclusions:**

Improved glycaemic control needs to be considered within the broader framework of managing noncommunicable diseases in a more integrated, coordinated and better financed healthcare system. Further studies are needed to operationalise the interventions identified for successful implementation.

## Background

The prevalence of diabetes mellitus is increasing worldwide, with the number of affected adults almost having quadrupled between 1985 and 2014 [1]. The African region has the greatest predicted increase in the number of people with diabetes of all International Diabetes Federation (IDF) regions – expected increase of 129% to 55 million people by 2045 [2]. In the Democratic Republic of Congo (DRC), the trend of increasing prevalence of diabetes is clear, but better organisation of health data is needed to assess the true extent of the problem. The latest estimates from the IDF showed a 5.8% prevalence of diabetes among adults [3] with disparities between urban and rural areas, as well as between the western and eastern parts of the country [4,5].

One of the major problems in the management of diabetes is the difficulty to achieve glycaemic control, which is essential for the prevention of complications and thus for the preservation of the quality of life of patients [6]. Complications of diabetes result in increased costs of care for patients, society, and the health care system [7].

Controlling blood sugar levels is a complex phenomenon. Its complexity is primarily due to the existence of numerous factors that determine it. A literature review enabled us to develop a conceptual model based on the Chronic Care Model [8]. The factors were grouped into the following categories: policy, healthcare system, community, and patients and their families [9–12]. Knowledge of the factors acting in a given environment is essential to determine the interventions needed to remedy the situation [13].

The complexity of glycaemic control also lies in the fact that there are many interventions identified to improve glycaemic control. Among the consensuses on the management of hyperglycaemia in type 2 patients, the one continually updated and steered by the American Diabetes Association (ADA) and the European Association for the Study of Diabetes (EASD) remains the usual reference and has advocated therapeutic education, hygienic and dietary measures, and patient-centred care, in particular taking into account patients’ preferences in the provision of care [14,15]. In 2019, a team of diabetes experts from sub-Saharan Africa, taking into account the difficulties encountered in managing diabetes in Africa, in particular the cost and availability of medicines, proposed an algorithm for initiating and intensifying diabetes treatment in sub-Saharan Africa [15]. In DRC, the latest Ministry of Health standards for diabetes treatment were issued in 2012 and have not been updated [16].

Still, worldwide, only half of patients with type 2 diabetes achieve good glycaemic control [17]. In Africa, poor control is common, and, in the DRC, recent studies have shown similar worrying results with only 16% to 32% of the patients with type 2 diabetes having controlled glycaemia [18].

In our setting, the need for better diabetes care and management of hyperglycaemia is still crucial. There is no framework for improving glycaemic control in Kinshasa, DRC. We conducted a series of studies to determine the magnitude and drivers of poor glycaemic control to inform a potential framework to address the poor glycaemic control in the Kinshasa DRC. So the Delphi work was to solicit the views of experts in the field based on the conducted studies’ findings to come up with a recommended framework to optimise glycaemic control in DRC.

## Methods

### Study design

In situation where there is insufficient or conflicting or overload information, consensus methods provide another means of synthetising information [19]. Consensus strategies create structured environments allowing the experts to be given the best available information on an issue and to provide more justifiable and credible solutions [19]. Two consensus methods are commonly used in health services research, the Delphi process and the nominal group technique [20]. Delphi process is the one that is commonly used to develop guidelines by healthcare professionals [20]. Thus, the Delphi consensus method is the best approach to develop a policy framework considering the multitude of factors associated with blood glucose control, the number of possible interventions, some with contrasting results, and the socio-economic and cultural peculiarities of Kinshasa, DRC [20].

**Figure 1.**
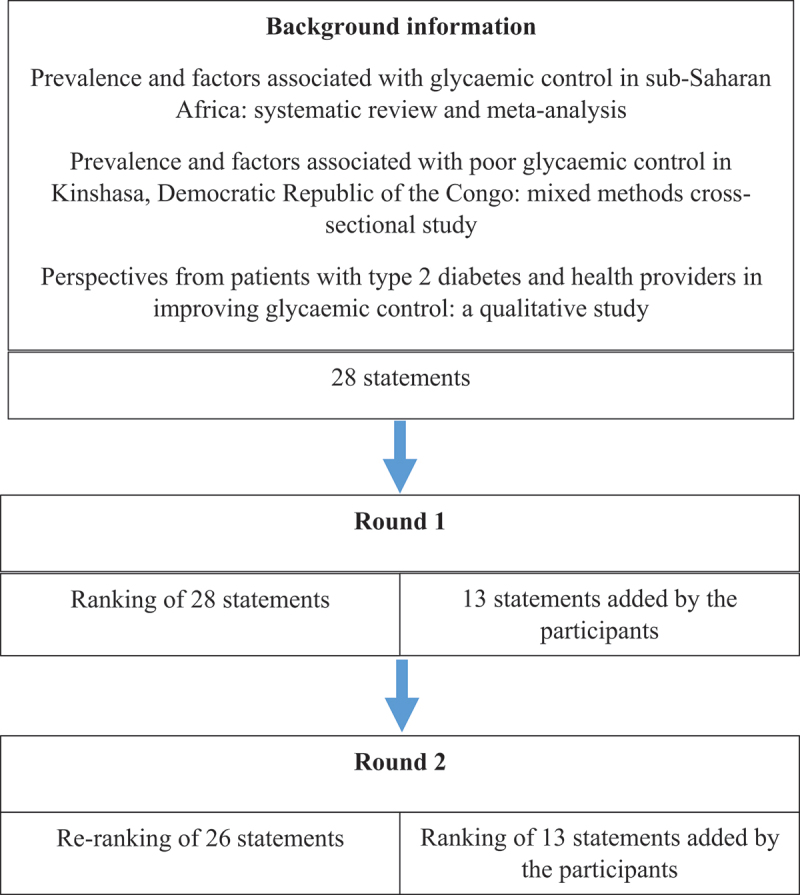
Description of the Delphi study process.

This was a two round, electronic, and anonymous Delphi study seeking policy consensus from important stakeholders in diabetes management in Kinshasa to develop a package of interventions for improving glycaemic control. The Delphi study is part of a larger project aimed at developing a framework for improving glycaemic control for patients with type 2 diabetes in Kinshasa, Democratic Republic of Congo, the protocol of which has been published [8]. Previously in the project, factors associated with glycaemic control in sub-Saharan Africa were determined in a meta-analysis [10]. Additionally, the factors associated with poor glycaemic control in Kinshasa, DRC were determined in a cross-sectional study and substantiated with findings from qualitative studies of the perspectives of patients and caregivers on the factors that affect glycaemic control and proposed interventions in Kinshasa. Informed by these studies, experts were brought together to define a package of interventions to improve glycaemic control among type 2 patients in the Democratic Republic of Congo through consensus. The process of the Delphi study is summarised in Figure 1. This Delphi study is reported according to the Guidance on Conducting and Reporting Delphi Studies (CREDES) [21].

### Context

The proposed interventions are for patients with type 2 diabetes in Kinshasa, the capital city of the DRC. The country is a low-income country with a population of 95,894,118 inhabitants [22]. In 2019, the country spent 4% of its Gross Domestic Product in health expenditure [23]. In 2022, about 64% of the population were living with less than 2.15 $US a day [22]. Most of the population are not covered by health insurance and have to rely on out-of-pocket payments. Kinshasa has a population of approximately 15 million within an area of 9 965 square kilometres [24]. In recent years, the population of Kinshasa is growing fast due to a rural exodus resulting from the destruction of economic infrastructure of the surrounding provinces. The care for diabetes patients is mainly organised via the Kinshasa Primary Care Network uniting health structures belonging to the Catholic Church and the Salvation Army [25]. The other facilities organising diabetes care are privately owned and some State health structures. The problem in this context is that there is no real integration or control of activities, with each player trying to find its own way with whatever resources it can muster. The national diabetes programme is not yet really effective on the ground. Data on the impact of diabetes are not well organised.

### Subjects

The Delphi study included representatives of stakeholders in diabetes management in Kinshasa, DRC. Two approaches were used to select the experts. The first was to contact the heads of institutions involved in diabetes and ask them to nominate representatives for the Delphi study. In the second approach, individuals known for their expertise in diabetes were contacted. We selected six health services managers, eight clinicians, seven representatives from universities, nine members of the staff of the National Program against Diabetes, and six people from non-government organisations working on diabetes in Kinshasa and in Africa. The clinicians were persons qualified in the clinical practice of medicine, namely five doctors or three nurses, and taking care of diabetes; managers were individuals in charge of a healthcare structure (hospital or health centre) or a group of healthcare structures organising diabetes care; representatives of universities were diabetologists or internal medicine specialists; NGO partners were all the persons within organisations participating in the management of diabetes by providing financial or technical support; representatives from the Ministry of Health were individuals working in the National Program of diabetes. The participants were purposively selected by the research team – principal researcher and supervisors – based on their interest in the subject and expertise in diabetes control. Contact was made with the structures organising or providing support for diabetes care to ensure that participants were experts in the issue; known experts in diabetes management were contacted directly through e-mail, telephone and WhatsApp.

All the participants had an age ≥18 years; experts were local and international experts with preference given to those who currently or previously worked in the DRC, and those with known expertise in diabetes. The exclusion criteria was conflict of interest in study participation.

Thirty-six participants were included in the first round of the Delphi from a total of 62 experts invited, representing a response rate of 58.1%. In the second round of the 36 participants who took part in the first round, two dropped out, and the response rate was of 94.4%.

### Data collection

E-mail was used to send an information document describing the purpose and objectives of the study to eligible people. They were requested to agree electronically to participate in the study. Once they accepted to participate in the study, a second e-mail informed them of the results of previous sub-studies of this project.

This Delphi study included two rounds. In the first round, each stakeholder was asked to use a 4-point Likert scale to indicate by how much he/she agreed or disagreed with the statements constructed based on the findings of previous studies in sub-Saharan Africa and Kinshasa on factors associated with glycaemic control and perspectives for improving glycaemic control. The questionnaire also comprised open questions for the participants to suggest additional interventions for improving glycaemic control or to comment on the proposed interventions. The questionnaire was designed on Redcap 13.4.13 [26] and shared with the participants through e-mail and WhatsApp. The questions were constructed using a 4-point Likert scale (‘strongly agree’, ‘agree’, ‘disagree’, and ‘strongly disagree’).

The participants’ responses in the first round were used to perform an initial assessment of the extent to which they agreed with the recommendations. At the end of the first round, the researcher summarised the rankings from the participants and their motivations and used these for the second round. Only the recommendations that were strongly agreed to by the participants and those suggested by the participants were used in the second round. The reliability of the survey tool used in the second round was assessed using the Cronbach’s alpha coefficient; the result was 0.97 indicating high internal consistency.

In this second round, the experts were asked to re-rank the statements after reconsidering their opinions or judgements in light of other group members’ responses and/or again to motivate their choice or suggest other interventions. No new themes were identified by the participants. For the majority of participants, the positions or responses did not vary substantially from those expressed in the first round. Their ratings consisted of assessing each intervention again according to a 4-point Likert scale (‘strongly agree’, ‘agree’, ‘disagree’, and ‘strongly disagree’). At the end of the second round, the researchers sorted a list ranking the remaining interventions along with minority opinions achieving consensus. Expert contributions were anonymous.

### Data analysis

For each round and for all proposed interventions, the frequencies of all responses of the 4-Likert were summarised to determine the mode. The means and standard deviations were used to describe group responses to each statement. For each statement, the overall interpretation used was: 4.00–3.00: strongly agree; 2.99–2.00: agree; 1.99–1.00: disagree; 1.00–0.99: strongly disagree [27,28].

At the end of a round, only recommendations with an average score above 3.00 should be considered for the next round. Between rounds, the data were analysed to identify convergence and change of experts’ opinions or judgements.

Consensus was defined as ˃70% of experts agreeing/strongly agreeing or disagreeing/strongly disagreeing with a statement in round 2. This level of agreement has been used in previous studies [29,30]. Stability of consensus was considered to have been reached if the between round group responses varied by ≤10%.

## Results

Table 1 presents the characteristics of the participants.

**Table 1.** Characteristics of the participants of the Delphi study, *n* = 36.

	Categories	Number	Percentage (%)
Age (years)			
	<40	14	38.9
	40–65	20	55.5
	≥65	2	5.5
Sex			
	Male	28	77.8
	Female	8	22.2
Role			
	Clinician	8	22.2
	Manager	6	16.7
	National Program Against Diabetes	9	25.0
	University	7	19.4
	Non-Government Organization	6	16.7
Background			
	Physician	26	72.2
	Nurse	3	8.3
	Economist	4	11.1
	Pharmacist	1	2.8
	Political Scientist	1	2.8
	Lawyer	1	2.8
Expertise (years)			
	<10	16	44.4
	10–19	13	36.1
	≥20	7	19.4
Location			
	National	31	86.1
	International	5	13.9

Five domains of statements were identified for improving glycaemic control. Table 2 presents a summary of grouped statements by domain from the Delphi study.

**Table 2.** Summary of grouped statements by domain.

	Number of statements in each domain		Proportion of statements where consensus was reached	
	Round 1	Round 2	Round 1	Round 2
Strengthening the healthcare system for better diabetes care	14	17	13	17
Enhancing the awareness of diabetes among the population/patients	3	5	3	5
Alleviating the financial burden of diabetes	4	9	4	9
Enhancing the adoption of lifestyle modifications in our setting	4	5	3	5
Reducing the proportion of undiagnosed patients with diabetes	3	3	3	3
Total	28	39	26	39

From the 28 initial statements, the participants agreed on 26 statements (92.9%) and two were excluded (7.1%). For the second round, these 26 statements were added to 13 additional recommendations suggested by the participants in the first round, a total of 39 statements were submitted to the experts and all reached consensus.

Table 3 describes in detail the assessment of each statement throughout the two rounds of the Delphi study. In the first round, ‘creating food banks’ and ‘implementing gatekeeping on prescriptions to improve adherence to evidence-based guidelines’ reached 55.9% and 69.7%, respectively, and were excluded. The participants also suggested 13 additional recommendations that were added to the second round of the Delphi study. All the statements reached the consensus (100%). The stability of the consensus was assessed for 26 of 39 statements (%); only one of the 26 statements had varied by more than 10%.

**Table 3.** Analysis of the responses to statements throughout the Delphi process.

		First round statistics (*n* = 36)				Second round statistics (*n* = 34)				
		Responses (%)				Responses (%)				
Domains	Strategies	Agreed/ strongly agreed	Disagree/ strongly disagree	Mean***	Standard deviation	Agreed/ strongly agreed	Disagree/ strongly disagree	Mean***	Standard deviation	Stability (%)
Strengthening the healthcare system for better diabetes care	Set up a diabetes primary care team	100.0	0.0	3.7	0.4	97.1	2.9	3.6	0.6	2.9
	Equip the health structures with basic equipment	100.0	0.0	3.7	0.4	97.1	2.9	3.8	0.6	2.9
	Set up an effective drug delivery system	94.1	5.9	3.5	0.6	100.0	0.0	3.6	0.7	5.9
	Develop a diabetes management protocol	91.7	8.3	3.6	0.6	97.1	2.9	3.7	0.6	5.4
	Augment geographical access to care	100	0.0	3.6	0.5	94.1	5.9	3.6	0.7	5.9
	Reduce waiting time for access to care	97.1	2.9	3.5	0.6	91.2	8.8	3.3	0.7	5.9
	Technical supervision	94.3	5.7	3.5	0.6	94.1	5.9	3.5	0.7	0.2
	Adopt glycosylated haemoglobin for glycaemic control	91.2	8.8	3.3	0.6	94.1	5.9	3.4	0.7	2.9
	Implement ‘gatekeeping’ on prescriptions to improve adherence to evidence-based guidelines*	69.7	30.3	2.9	0.7					
	Strengthen the National Diabetes Control Program**					97.1	2.9	3.7	0.6	
	Develop individual care for patients	94.2	5.8	3.5	0.6	97.1	2.9	3.5	0.7	2.9
	Adhering to evidence-based clinical guidelines	100.0	0.0	3.6	0.5	94.1	5.9	3.4	0.7	5.9
	Innovative approach to patients	100.0	0.0	3.5	0.5	97.1	2.9	3.4	0.7	2.9
	Support self-management of the patients	85.7	14.3	3.3	0.8	91.2	8.8	3.5	0.7	5.5
	Identify patients at risk of poor glycaemic control	100.0	0.0	3.7	0.4	94.1	5.9	3.5	0.7	5.9
	Educate the family and communicate the objectives of the family with the patient’s consent**					97.1	2.9	3.6	0.7	
	Identify patients with poor glycaemic profile**					94.1	5.9	3.5	0.8	
	Send text messages to remind patients to take their medicines or to respond to follow-up visit**					85.3	14.7	3.3	0.8	
Enhancing the awareness of diabetes among the population/patients	Develop radio/TV spots on diabetes	100.0	0.0	3.7	0.5	94.1	5.9	3.5	0.7	5.9
	Distribute information leaflets on diabetes	77.1	22.9	3.1	0.8	88.2	11.8	3.3	0.8	11.1
	Send text/videos through social media	97.2	2.9	3.5	0.6	91.2	8.8	3.3	0.8	6.0
	Community-based awareness activities in churches, schools, and open spaces (markets)**					97.1	2.9	3.7	0.6	
	The train and involve community health workers in the sensitization of the population**					97.1	2.9	3.7	0.6	
Alleviating the financial burden of diabetes	Implement universal health coverage	100.0	0.0	3.7	0.5	97.1	2.9	3.7	0.6	2.9
	Promoting health insurance	88.2	11.8	3.5	0.7	97.1	2.9	3.5	0.7	8.9
	Encourage the creation of health mutuals	97.1	2.9	3.6	0.5	97.1	2.9	3.6	0.7	0.0
	Encourage family involvement and support	85.3	14.7	3.2	0.8	94.1	5.9	3.6	0.7	8.8
	Partner with pharmaceutical industry to avail good quality generic drugs at affordable cost**					94.1	5.9	3.5	0.8	
	Improve the standard of living of the Congolese population by granting an acceptable minimum wage to the lowest paid workers so that they are able to contribute to or support the mutual insurance system**					94.1	5.9	3.6	0.7	
	Promote flat-rate pricing for disease episodes or even monthly patient care at the center or hospital.**					97.1	2.9	3.5	0.7	
	Exempt imported equipment for diabetes care (insulin, strips, syringes …) from excise and import duties to reduce costs**					94.1	5.9	3.6	0.7	
	Allocation of a significant budget for chronic diseases**					97.1	2.9	3.5	0.7	
Enhancing the adoption of lifestyle modifications in our setting	Develop a suitable exercise plan for patients considering their occupations	91.4	8.6	3.5	0.7	94.1	5.9	3.4	0.7	0.0
	Encourage realistic dietary plans that considers affordability	97.1	2.9	3.8	0.5	97.1	2.9	3.6	0.6	0.0
	Create food banks*	55.9	44.1	2.7	0.8					
	Providing accurate information on recommended diet and exercise	97.1	2.9	3.6	0.5	97.1	2.9	3.7	0.6	0.0
	Promote primary prevention using policies that target marketing and sales of of tobacco, alcohol, sweetened beverages, and fast foods**					82.2	17.8	3.3	0.8	
	Open gymnasiums and encourage healthy and balanced eating**					88.2	11.8	3.2	0.7	
Reducing the proportion of undiagnosed patients with diabetes	Implement opportunistic screening at primary care level	94.4	5.6	3.7	0.6	97.1	2.9	3.7	0.6	2.7
	Promote cases finding among in high-risk population e.g. patients’ families	88.2	11.8	3.2	0.6	88.2	11.8	3.3	0.8	0.0
	Mass screening during diabetes international day	88.6	11.4	3.3	0.8	94.1	5.9	3.5	0.7	5.5

Legend:  *Strategy excluded after the first round; **strategy added after the first round; ***scale range: for each statement, the overall interpretation is: 4.00–3.00: strongly agree; 2.99–2.00: agree; 1.99–1.00: disagree; 1.00–0.99: strongly disagree.

## Discussion

This Delphi study aimed to build a policy consensus on recommendations for improving glycaemic control in Kinshasa, DRC. The study identified five domains containing 39 statements: strengthening healthcare system for better diabetes care, enhancing the awareness of diabetes among the population/patients, alleviating the financial burden of diabetes, enhancing the adoption of lifestyle modifications in our setting, reducing the proportion of undiagnosed patients with diabetes, and helping healthcare providers to act for improving glycaemic control.

### Strengthening the healthcare system for better diabetes care

Improving glycaemic control among patients with type 2 diabetes cannot be realised without strengthening of the health system. The WHO framework characterised a well-functioning health system as one that puts together trained and motivated health workers, a well-maintained infrastructure, a reliable supply of medicines and technologies, backed by adequate funding, strong health plans, and evidence-based policies [31]. These elements have been identified in the recommendations made by the experts during the current Delphi study. Concerning healthcare providers, the recommendations of this Delphi study highlighted the benefits of establishing multidisciplinary care teams for better diabetes care at the primary health care level. Such multidisciplinary teams have been linked to better outcomes, including good glycaemic control among patients with type 2 diabetes [32]. In this multidisciplinary team, task shifting should be strongly considered considering the shortage of qualified and specialised healthcare providers in Kinshasa. Interventions led by nurses and pharmacists in Low- and Middle-Income Countries (LMICS) with relatively low physician density are effective in the management of diabetes [33]. In a realistic and cost-effective way, improving diabetes care has to be considered in the broader framework of managing noncommunicable diseases in the primary healthcare level [34,35]. For these changes to occur, diabetes and more noncommunicable diseases should be taken at their just value by the policymakers. The Ministry of Health through the National Program against diabetes should provide data that translates the burden of diabetes and advocate for prioritisation.

The Government should engage itself to provide more funding to health to better prepare the health system to face noncommunicable diseases. The Ministry of health should also ensure the development of clear guidelines for the management of diabetes, which take into account the specificities of diabetes in our setting. The Ministry of Health and the National Program against Diabetes should also warrant that healthcare staff are trained, motivated and assigned to diabetes management centres to provide all the package required for the care for patients with diabetes.

Healthcare providers must be trained on this guideline and formative supervision and oversight by more qualified clinicians of the activities should help to improve the quality of care. In the training of the healthcare providers, caution must be taken to consider the development of a patient-centred approach [36], and to ensure prevention of poor glycaemic control by identifying high-risk patients.

Strengthening of the healthcare system should also include enabling a bidirectional referral pathway that allows for escalated/more complex care at highly specialised level (for patients who are uncontrolled at primary healthcare (PHC) and de-escalated care to PHC level for follow up when such patients become stabilised) [37].

Funding is critical in implementing the changes required in the structure and processes of care for better diabetes management [38]. There is a need to ensure, as outlined in the Global Diabetes Compact, that everyone with diabetes should have access to equitable, comprehensive, affordable, and quality treatment and care [39]. The government has just started the process of universal health coverage, but diabetes is not yet covered due to negative financial constraints. Extending universal health coverage to diabetes will allow vulnerable and disadvantaged individuals access to equitable care [40].

### Alleviating financial burden of diabetes

Empiric evidence shows that better socioeconomic conditions are associated with improved glycaemic control [41]. The Government should ensure that the war against poverty is effective as it could allow persons with diabetes to have their purchasing power to grow in order to face the charges of diabetes. The Government should also provide social protection to vulnerable categories of persons with diabetes through the extension of universal health coverage to noncommunicable diseases. One way to reduce the burden of diabetes is also to help persons with diabetes access to affordable medications. A partnership with the pharmaceutical sector mainly private could help in this purpose. The government could reduce taxes on medications and supplies for the care of diabetes and other non-communicable diseases, with a view to lowering their cost on the market.

In the search for a reduction in the cost of health care, the integration of a regulation of prescriptions could be beneficial as it has been dismantled in other settings [42]. The panel of experts did not opt for this recommendation by a very narrow margin of consensus. The lack of health insurance may have been a reason for rejecting this measure. Nevertheless, technical supervision may have as one of its objectives the monitoring of prescriptions. They also rejected the creation of food banks on the grounds that the healthcare system is suffering from budgetary constraints and that these banks could require far too much financial resources to organise.

In the healthcare system level, integrating PEN-Plus could be a cost-effective approach [35]. The DRC has not yet incorporated this model. There is a need to move towards the implementation of PEN and PEN-Plus in our setting. In the long term, the population must be financially empowered to face the costs of diabetes care by prepaid payments – health insurance or health mutual. Out-of-pocket payment for health care currently represents a serious barrier to healthcare access in Kinshasa and could lead to impoverishment [43].

At the level of the relatives, one may note that family represent sometimes the sole source of support for the patients. The healthcare providers must be encouraged to develop a family-oriented care approach for patients to increase the involvement of the patients’ relatives in their care [44]. Family support has been linked to better health outcomes [44]. They must develop a family orientated care to ensure that the relatives understand the diabetes disease to ease their support to the sufferer

### Enhancing the awareness of diabetes

Raising the awareness of the population will also contribute to acceptable health seeking behaviours [45]. Participants pointed out that people in the community do not read much, unsurprisingly because most are not highly educated; they are more attracted to plays on television. The national programme against diabetes should organise awareness campaigns for these diseases. As part of this campaign, we need to take account of the need to get messages across via the plays that are popular with the public. Community-based campaigns in public spaces, churches, and schools have been seen as an appropriate approach in our environment.

### Enhancing the adoption of lifestyle modifications

Lifestyle interventions have been found to be effective for glycaemic control in low- and middle-income countries [46]. For the persons living with diabetes in our setting, the critical point is to integrate these changes in the daily activities. Advocating for the integration of the practice of exercise in workplace will be of greater usefulness. Policies that target marketing and sales of tobacco, alcohol, sweetened beverages, and fast foods could also be applied to try to control their use by the public. As far as diet is concerned, it is essential to define appropriate diets that take account of patients’ sometimes low incomes. Operational research is needed to determine the best interventions for lifestyle change, taking into account the patient’s condition and purchasing power.

### Reducing the proportion of undiagnosed diabetes

Incorporating opportunistic screening of diabetes by targeting high-risk individuals could ease the management of diabetes at the time of diagnosis and delay the occurrence of complications. Opportunistic screening is one of the cost effectiveness way to screen for diabetes [47]. The healthcare facilities and their teams should be prepared and equipped to offer these activities.

### Strengths and limitations of the study

This is the first study in Kinshasa, Democratic Republic of the Congo that tried to build consensus on how to improve glycaemic control using a Delphi panel of experts [48]. This Delphi study was supported by a rich background of information from the findings of previous studies that identified the factors of glycaemic control in sub-Saharan Africa and Kinshasa and the perspectives from patients with type 2 diabetes and healthcare providers in improving glycaemic control.

The weakness of the study was that Delphi studies do not allow live discussions that could have increased the depth of engagement and potentially given rise to more ideas on the issue. The consensus concerned a specific situation and therefore may not be generalisable [49]. The high proportion of participants with medical background could have introduced a bias in the findings of this Delphi study.

## Conclusions

The study found that participants had a high level of consensus on how to improve glycaemic control among patients with type 2 diabetes in Kinshasa with most statements converging around five domains that focused on the need for a more integrated, coordinated and adequately financed healthcare system for better diabetes care. All of these statements were expressed within a broader framework of managing noncommunicable diseases. Further studies are needed to operationalise the interventions identified to determine their scalability, effectiveness, efficiency and acceptability.

## Paper context

Achieving good glycaemic control is a global challenge. The challenge is particularly acute in sub-Saharan Africa due to multiple barriers. This study identified a number of interventions for improving glycaemic control in a particular context, but which may be relevant in similar settings.
